# Case series of 12 *Bartonella quintana* endocarditis from the Southwest Indian Ocean

**DOI:** 10.1371/journal.pntd.0011606

**Published:** 2023-09-07

**Authors:** Ludivine Sarsiat, Thomas Garrigos, Linda Houhamdi, Olivier Dauwalder, Barbara Kuli, Eric Braunberger, Olivier Belmonte, Pierre-Edouard Fournier, Guillaume Miltgen

**Affiliations:** 1 Laboratoire de Bactériologie, CHU Félix Guyon, Saint-Denis, La Réunion, France; 2 UMR Processus Infectieux en Milieu Insulaire Tropical (PIMIT), CNRS 9192, INSERM U1187, IRD 249, Université de La Réunion, Saint-Denis, La Réunion, France; 3 CNR des Rickettsies, *Coxiella* et *Bartonella*, IHU-Méditerranée Infection, Marseille, France; 4 Plateau de Microbiologie Moléculaire Spécialisé et de Séquençage, Institut des Agents Infectieux, Centre de Biologie et Pathologie Nord, Hospices Civils de Lyon, Lyon, France; 5 Service de Maladies Infectieuses, CHU Félix Guyon, Saint-Denis, La Réunion, France; 6 Service de Chirurgie Cardio-thoracique, CHU Félix Guyon, Saint-Denis, La Réunion, France; 7 UMR Vecteurs—Infections Tropicales et Méditerranéennes (VITROME), Université d’Aix-Marseille, IRD, AP-HM, SSA, IHU-Méditerranée Infection, Marseille, France; 8 Centre Régional en Antibiothérapie (CRAtb) de La Réunion, France; University Hospital of Frankfurt, GERMANY

## Abstract

**Background:**

*Bartonella* spp. are fastidious bacteria frequently identified as the cause of blood culture-negative (BCN) endocarditis. However, *Bartonella* infections are difficult to diagnose in routine laboratory testing and their incidence is probably underestimated. We investigated the epidemiological and clinical features of *Bartonella* endocarditis cases diagnosed between 2009 and 2021 on Reunion Island (Southwest Indian Ocean).

**Method:**

We retrospectively included all patients diagnosed with *Bartonella* endocarditis at Reunion Island University Hospital during this period. Endocarditis was diagnosed on the basis of microbiological findings, including serological tests (IFA) and PCR on cardiac valves, and the modified Duke criteria. We used then the multispacer typing (MST) method to genotype the available *Bartonella* strains.

**Findings:**

We report 12 cases of *B*. *quintana* endocarditis on Reunion Island (83.3% in men, median patient age: 32 years). All the patients originated from the Comoros archipelago. The traditional risk factors for *B*. *quintana* infection (homelessness, alcoholism, exposure to body lice) were absent in all but two of the patients, who reported head louse infestations in childhood. Previous heart disease leading to valve dysfunction was recorded in 50% of patients. All patients underwent cardiac valve surgery and antimicrobial therapy with a regimen including doxycycline. All patients presented high C-reactive protein concentrations, anemia and negative blood cultures. The titer of IgG antibodies against *Bartonella* sp. exceeded 1:800 in 42% of patients. Specific PCR on cardiac valves confirmed the diagnosis of *B*. *quintana* endocarditis in all patients. Genotyping by the MST method was performed on four strains detected in preserved excised valves and was contributive for three, which displayed the MST6 genotype.

**Conclusions:**

*Bartonella quintana* is an important cause of infective endocarditis in the Comoros archipelago and should be suspected in patients with mitral valve dysfunction and BCN from this area.

## Author summary

*Bartonella quintana*, best known as the cause of trench fever in soldiers during the First World War, can also cause endocarditis in humans. It is transmitted predominantly by the feces of body lice. After entering the bloodstream, it may become localized in damaged valves or tissues. Nevertheless, *B*. *quintana* remains an uncommon cause of infective endocarditis with few cases reported worldwide. However, the number of reported cases of this pathogen has recently increased mostly in refugee camps and among underhoused individuals in Europe and the USA.

We describe here 12 cases of *B*. *quintana* endocarditis in patients from Mayotte/the Comoros archipelago in the Southwest Indian Ocean. We highlight the presence of this pathogen in patients from this region characterized by high rates of poverty and population exchanges with Southern Africa.

Genotyping analysis on three strains from this cohort revealed the presence of a single genotype, different from those observed in Europe and Africa, suggesting the presence of a single *B*. *quintana* genotype in the Comoros archipelago, or a potential common source of contamination.

This study sheds light on the etiology of this neglected form of endocarditis and should raise local awareness of this disease.

## Introduction

*Bartonella quintana* is a small Gram-negative bacillus from class Alphaproteobacteria. These fastidious bacteria are generally transmitted by arthropod vectors, predominantly in the feces of body lice, and can cause various human infections. *B*. *quintana* was initially identified as the cause of trench fever in soldiers during World War I, but now mostly affects people living in poor sanitary conditions worldwide [1]. This re-emerging bacterium causes bacteremia and infective endocarditis (IE) in homeless and alcohol-dependent individuals in Europe and America. It is now often referred to as “urban trench fever” [2]. *B*. *quintana* can also cause bacillary angiomatosis and peliosis hepatitis in immunocompromised patients [3].

The first case of *B*. *quintana* endocarditis was reported in 1993 [4], and several hundred cases have since been described, mostly in Europe [5]. Other *Bartonella* species have also been implicated in endocarditis, notably *B*. *henselae*, and occasionally *B*. *elizabethae*, *B*. *vinsonii subsp*. *berkhoffii*, *B*. *vinsonii subsp*. *arupensis*, *B*. *koehlerae*, *B*. *alsatica* and “*Candidatus* Bartonella mayotimonensis” [6–13]. Published epidemiological data suggest that the prevalence of *Bartonella* endocarditis follows a European-African gradient, with prevalence increasing from north to south [5, 14]. *Bartonella* species are facultative intraerythrocytic bacteria with slow replication that are known to cause blood culture-negative endocarditis (BCNE), accounting for up to 28.4% of cases [5, 15]. They are, therefore, no readily detected in routine diagnostic procedures if their presence is not initially suspected, rendering diagnosis difficult [5].

Very few data are available concerning *B*. *quintana* endocarditis in South-East Africa and the Indian subcontinent, and no case has ever before been reported in the Southwest Indian Ocean area. Moreover, very few studies on *B*. *quintana* endocarditis have reported data for strain genotyping. Such analyses are useful for epidemiological investigations aiming to determine the source of the infection [16, 17].

The aim of this study was to review the epidemiological and clinical data, and outcomes of the documented cases of *B*. *quintana* endocarditis diagnosed on Reunion Island from 2009 to 2021, and to discuss possible links between cases or a common source of infection.

## Methods

### Ethics statement

Since this was a retrospective study based on archived patient records, it falls into the category of research not involving the human person (article L.1121-1 of the French Public Health Code, deliberation no. 2018–155 of the French Data Protection Authority (CNIL)). In addition, this research was performed in accordance with the Declaration of Helsinki. Consequently, committee approval was not required.

### Patient recruitment

All definite cases of *Bartonella* endocarditis diagnosed at Reunion Island University Hospital from 2009 to 2021 were retrospectively included in this study. All patients diagnosed with infective endocarditis (IE) on Reunion Island are treated at this hospital. For each case, medical records were analyzed and various data were extracted, including age, sex, year of diagnosis, country of residence, medical history, clinical presentation, presence of fever, possible exposure, suspected infection pathway, affected valve, surgical intervention, determination of C-reactive protein and hemoglobin levels, antibacterial treatment and outcome.

### Case definition

The diagnosis of definite *Bartonella* endocarditis is based on at least two major Duke score criteria: *(i)* echocardiogram positive for endocarditis, as defined in the modified Duke criteria [18, 19] and *(ii) Bartonella*-positive PCR on infected cardiac tissue, as defined in the recent update of the modified Duke criteria [18, 20, 21]. According to the guidelines followed in our medical laboratory [19], blood is collected for culture in cases of suspected endocarditis and incubated for 15 days. In cases of negative blood culture, serological testing is performed for *Bartonella* spp. and *Coxiella burnetii*. PCR analysis is also performed in patients undergoing valve surgery.

Serological analyses were based on immunofluorescence assays (IFA), as previously described [22], to determine the titers of antibodies directed against *B*. *henselae*, *B*. *quintana* (*Bartonella* IFA-Focus Diagnostics) and *Coxiella burnetii* (Q Fever IFA-Focus Diagnostics). PCR analyses were performed on bacterial DNA extracted from surgically excised valves. Two PCR were performed on an Ingenius instrument (ELITech Groupe France), targeting *(i) Bartonella* spp. (16S rRNA gene, Bartonella PROGENIE kit-Orgentec) and *(ii) B*. *quintana (ribC* gene, homemade PCR).

### Genotyping

We used the multispacer typing (MST) genotyping method to identify and compare *B*. *quintana* strains. The culture of *Bartonella* spp. is challenging, and the MST approach makes it possible to circumvent the difficulties of culture, by making it possible to use directly extracted DNA, with sufficient resolution to discriminate between *Bartonella* clones. The MST method for *Bartonella* was set up by Foucault *et al*. through the amplification and sequencing of 34 spacers from a collection of 71 isolates, and can be used to characterize several genotypes based on the combined single-nucleotide polymorphism (SNP) profiles of two spacers (336 and 894) [23]. This method is suitable for use in epidemiological and evolutionary studies. In this study, DNA was extracted from preserved *B*. *quintana* PCR-positive cardiac samples, when available. The analysis was performed by the French National Reference Center (IHU Méditerranée Infection, Marseille, France).

## Results

### Characteristics of the patients and epidemiological data (Table 1)

In total, 12 cases were included in the study. Most of the infected patients were male (83.3%) and the median age was 32 years (IQR: 23.75–40.5 years). The recorded sites of residence of the patients were: Mayotte (*n* = 6, 50%), Comoros (*n* = 5, 41.7%) and Reunion Island (*n* = 1, 8.3%). Five of the six patients from Mayotte had recently traveled to or originated from Comoros. The only patient living on Reunion Island had also recently moved there from Mayotte (Table 1). None of the patients was immunocompromised or homeless, although information about living conditions was not available for all patients. However, three patients (25%) reported living in poor socioeconomic conditions: sheet metal house, clay ground and absence of running water. The population of Comoros is mostly Muslim, so alcohol consumption is uncommon. None of the patients had an alcohol use disorder. A history of contact with head lice was reported for only two patients (16.7%), and two patients, both farmers, reported contact with animals.

**Table 1:** Demographic characteristics of the 12 patients with documented *B*. *quintana* endocarditis.

Pat. No.	Sex	Origin	Year of initial presentation	Age at initial presentation (years)	Medical history	Possible exposure, links to rural community
1	M	Comoros/Mayotte	2009	39	No relevant medical history*	U
2	M	Comoros/Mayotte	2009	21	Rheumatic heart disease	Head louse infestations in childhood, low-income group
3	F	Comoros	2009	24	Atrial septal defect	Head louse infestations in childhood, low-income group
4	M	Comoros	2010	19	No relevant medical history*	Low-income group
5	M	Mayotte	2013	46	Hypertension	U
6	M	Comoros/Mayotte	2013	38	No relevant medical history*	U
7	M	Comoros	2018	23	Rheumatic heart disease, hypertension, cardiac arrhythmia	Contact with animals (farmer)
8	M	Comoros/Mayotte	2018	27	Rheumatic heart disease, systolic ejection murmurs	U
9	M	Comoros	2019	45	Rheumatic heart disease	U
10	M	Comoros	2020	28	Rheumatic heart disease	U
11	F	Comoros/Mayotte	2020	36	No relevant medical history*	U
12	M	Reunion Island/Mayotte	2020	69	Hypertension	Contact with animals (farmer)

*: No relevant medical history connected with cardiac or infectious disease; F: female; M: male; U: unknown.

### Clinical features (Table 2)

Eight patients (66.7%) had fever at initial presentation. Heart failure was the main presenting form of endocarditis caused by *B*. *quintana* in our cohort, reported in 11 patients (91.7%). Heart failure was mostly due to valvular insufficiency (10/11 patients). Acute renal failure complicating severe chronic renal failure was observed in one of the 12 patients (8.3%). Embolic phenomena were recorded in seven patients (58.3%, Table 2), most frequently taking the form of splenic (3 patients, 25%) or intracranial mycotic aneurysms (2 patients, 16.6%). All patients had native valves during the first episode of IE. The mitral valve was affected in 10 patients (83.3%), four of whom also had aortic valve involvement, with one patient also having tricuspid valve involvement. In two patients (16.7%) only the aortic valve was involved.

**Table 2:** Clinical features of the 12 patients with documented *B*. *quintana* endocarditis.

Pat. No.	Presenting symptoms	Fever	Embolism	Affected valve(s)	Postoperative complications	Antibacterial treatment after diagnosis of *Bartonella* infection	Outcome
1	MI, DHF, cardiogenic shock, ARF	No	No	MIT	CRD	GEN (3 wk) + DOXY (6 wk)	MI, surgical replacement of mitral valve 7 years later
2	AI, MI, septic syndrome	Yes	Splenic	AO, MIT	None	GEN (2 wk) + AMX (4 wk) Then DOXY (4 wk) + RIF (4 wk)	3 months later: *Streptococcus mitis* sepsis and endocarditis 5 years later: *Streptococcus gordonii* endocarditis, aortic and mitral valve replacement surgery 7 years later: *Streptococcus sanguinis* sepsis
3	AGS, 8 mo shortness of breath, APE, cachexia, DHF	Yes	No	MIT, TRI	CRD, APE	GEN (3 wk) + DOXY (6 wk)	Favorable
4	4 mo AGS, dyspnea, MI	Yes	No	MIT	CRD, pneumothorax, non-fatal cardiorespiratory arrest 1 day after surgery	GEN (3 wk) + DOXY (6 wk)	Favorable Jugular vein thrombosis
5	Dyspnea exertional, AI, severe hypertension	No	No	AO	Hypertensive crisis	DOXY (4 wk) + RIF (3 wk)	Favorable Recurrent hypertensive crisis
6	Acute coronary syndrome, DHF, APE	Yes	Splenic Coronary Cerebral	AO	None	DOXY (6 wk)	Major heart failure sequelae
7	7 y dyspnea exertional, chest pain, liver pain, AGS, anorexia, cough, nausea, diarrhea, inguinal and jugular adenopathy	Yes	No	MIT	CRD	GEN (2 wk) + HCQ + DOXY (long term, because of *Coxiella burnetii* coinfection)	Favorable
8	Malaise and altered consciousness, SAH, hemodynamic deterioration, septic shock	Yes	Mycotic aneurysm	AO, MIT	None	AMX (2 wk) + GEN (2 wk) + DOXY (6 wk)	Persistent heart failure Chronic headaches
9	Severe headache, hemiplegia, SAH, AI, MI	Yes	Mycotic aneurysm	MIT	Mediastinitis	AMX + DOXY (6 wk)	Favorable
10	Several mo dyspnea, night cough, AI chest pain, acute coronary syndrome, undernutrition	Yes	Splenic	AO, MIT	Mediastinitis, cardiac tamponade	DOXY (3 mo)	Favorable 2 years later: mitral valve stenosis
11	Acute respiratory distress syndrome, APE, CRD, severe undernutrition	No	Suspected pulmonary and CVA	MIT	CRD	GEN (2 wk) + DOXY (3 mo)	CRD
12	Altered general state, 3 mo dyspnea exertional, leg swelling, AI, DHF, undernutrition	No	Cerebral	AO	CRD (requiring pacemaker implantation), ARF	RIF (2 wk) + DOXY (6 wk)	Favorable

AGS: altered general state; AI: aortic insufficiency; AMX: amoxicillin; AO: aortic valve; APE: acute pulmonary edema; ARF: acute renal failure; CRD: cardiac rhythm disorder; CTX: cefotaxime; CVA: cerebral vascular accident; d: day(s); DHF: decompensated heart failure; DOXY: doxycycline; GEN: gentamicin; HCQ: hydroxychloroquine; MI: mitral insufficiency; MIT: mitral valve; mo: month(s); RIF: rifampicin; SAH: subarachnoid hemorrhage; TRI: tricuspid valve; wk: week(s); y: year(s).

### Treatment and outcome (Table 2)

In this study, all patients received doxycycline for at least four weeks. Seven patients (58.3%) received concomitant gentamicin for at least two weeks. Two patients received rifampicin, and one received amoxicillin in place of gentamicin, in combination with doxycycline.

Cardiac valve surgery was performed in all patients. Significant postoperative complications occurred in eight patients: six suffered from cardiac rhythm disorders, and two had mediastinitis. One of the two cases of postoperative mediastinitis involved *Staphylococcus epidermidis*, whereas the causal organism was not documented in the other case. None of the patients died from IE, and none suffered a relapse of *B*. *quintana* IE. However, one patient experienced three episodes of other types of bacterial endocarditis and sepsis in the following months and years. One patient had to undergo a second operation to replace the mitral valve seven years after his initial IE. Two patients developed heart failure sequelae: follow-up heart scintigraphy on patient 6 showed an altered post-stress left ventricular ejection fraction one month after medical care, whereas patient 8 presented grade 1 aortic regurgitation and grade 2 mitral regurgitation three years after surgery.

### Diagnosis

All patients had *B*. *quintana* infection confirmed by molecular testing on a cardiac specimen. Blood cultures were performed and were negative for all patients. Serological testing was performed for 11 patients, and nine patients (75%) had antibody titers >1:100. The French National Reference Center considers IFA results to be positive if the IgG antibody titer is ≥1:100, and uses a titer of 1:800 as the cutoff for IE diagnosis [5]. Six patients (54.5%) had antibody titers >1:800. Serological results were negative for two patients (16.7%). Laboratory tests revealed high C-reactive protein levels (median: 61 mg/L, range: 14.3–189 mg/L) and anemia (median Hb: 9.35 g/dL, range: 7.6–11.1 g/dL) in all patients.

Echocardiography records were obtained for all patients. In Table 3, echocardiograms are described as TTE if transthoracic, TEE if transesophageal and ECHO if the type of echocardiogram was not specified. Vegetations were observed in 10 patients (83.3%).

**Table 3:** Diagnostic data for the 12 patients with documented *B*. *quintana* endocarditis.

Pat. No.	Blood culture	Positive *B*. *quintana*-specific PCR	Other positive PCR results	Serology results		CRP (mg/L)	Hb (g/dL)	Main echocardiogram findings *(vegetation size)*	Genotype (MST)
				*B*. *quintana*	*B*. *henselae*				
1	Negative	MIT			IgG: 4096 IgM < 12	33	9.7	TTE: vegetation on mitral valve	NP
2	Negative	AO			IgG: 256 IgM < 12	189	8.8	TTE: vegetation on aortic and mitral valves	NP
3	Negative	MIT TRI			IgG: 512 IgM < 12	40	8.2	TTE: vegetation on mitral valve *(15mm)*	NP
4	Negative	MIT			IgG: 1024 IgM < 12	58	8.6	ECHO: vegetation on mitral valve	NP
5	Negative	Vegetations, AO		IgG: 64		74	10.6	TEE: vegetation on aortic valve *(8x5*.*5mm)*	NP
6	Negative	AO		IgG: positive		64	9.8	ECHO: multiple vegetations on aortic valve *(2-8mm)*	NP
7	Negative	MIT		IgG: 512 IgM < 12	IgG: 2048 IgM < 48	124.8	8.6	TTE: valve mitral insufficiency, no vegetations seen	NI
8	Negative	Pericardial membrane, mitral vegetation	*C*. *burnetii* (mitral valve) *L*. *lactis* (pericardial membrane, pericardial fluid)	IgG: 1280	IgG: 2560	14.3	9	ECHO: vegetations on mitral and aortic valves *(7x2mm)*	6
9	Negative	MIT				13.8	10	TTE: multiple vegetations on mitral valve, aortic insufficiency *(12-16mm)*	NP
10	Negative	AO, aortic vegetation		IgG: 1280	IgG: 2560	84	9.8	TEE: vegetations on aortic valve, aortic and tricuspid insufficiency *(10mm)*	6
11	Negative	MIT		IgG: <320	IgG: <320	120.6	11.1	TTE, TEE: hyperechoic mass on mitral valve	NP
12	1 positive culture (*A*. *junii*, *K*.*p*, *E*.*coli*) Likely contamina-tion	AO		IgG: 2560	IgG: 2560	28	7.6	TTE, TEE: vegetation on aortic valve, *(15x13mm)*	6

*A*. *junii*: *Acinetobacter junii*; AO: aortic valve; *C*. *burnetii*: *Coxiella burnetii*; ECHO: echocardiogram; *E*.*coli*: *Escherichia coli*; Hb: hemoglobin; *K*.*p*: *Klebsiella pneumoniae*; *L*. *lactis*: *Lactococcus lactis*; MIT: mitral valve; NI: not informative; NP: not performed; TRI: tricuspid valve; TEE: transesophageal echocardiogram; TTE: transthoracic echocardiogram.

All patients required valve replacement, due to cardiac valve destruction or insufficiency. The results of PCR for *B*. *quintana* on the DNA extracted from cardiac valve tissue or vegetations were positive for all 12 patients.

### Sequence typing

Residual biological material (cardiac specimens or serum samples) was available for sequence typing for only four of the 12 cases. MST analysis was informative in three of these cases. It was performed on three cardiac samples, from patients 8, 10 and 12. Spacers 336 and 894 were amplified by PCR and their nucleotide sequence was determined. All three strains were found to have the same MST profile, with type 1 as the sequence profile for spacer 336, and type 5 for spacer 894. Based on this combination of sequence types, we classified the three isolates as genotype MST 6 (S1 Table).

## Discussion

We report here a first series of 12 cases of *B*. *quintana* infective endocarditis occurring in the Southwest Indian Ocean area from 2009 to 2021.

Cases of *Bartonella* infective endocarditis have been reported worldwide, mostly in the Americas and Europe with bacteria of this genus accounting for 0 to 4.5% of infective endocarditis cases. The most frequently reported species in such cases is *B*. *quintana* [5, 15, 24–44]. However, only a few cases from other countries have been reported to date [15, 44–54], and the incidence of *Bartonella* infective endocarditis in the Southwest Indian Ocean remains unknown.

The Cardiothoracic Surgery Department of Reunion Island University Hospital is the reference center for the diagnosis and treatment of endocarditis in the Southwest Indian Ocean area, providing care for all patients from this region. Based on the data collected at Reunion Island University Hospital for 2020, the prevalence of *Bartonella* endocarditis was 3.1% among the documented cases of IE. However, the prevalence of *Bartonella* IE in this region is almost certainly underestimated, as only the most serious clinical cases benefit from medical evacuation from Mayotte to Reunion Island, as demonstrated by the high rates of heart failure and valve damage in our series of patients (all of whom required surgery).

Our findings confirm published data indicating a male preponderance and low median age [41, 55–57]. Clinical presentation was similar to that for other types of subacute bacterial endocarditis. However, severe acute symptoms (acute pulmonary edema, acute coronary syndrome, subarachnoid hemorrhage) were often present at initial presentation, as previously reported [15, 45]. This finding may be explained by the subacute pathophysiology of *Bartonella* IE, and by late diagnosis and care, as access to healthcare is often very difficult in poor areas, such as Mayotte and Comoros. It is important to remind that patients in this cohort are transferred from Mayotte/Comoros to Reunion Island at an advanced stage of the disease, thus presenting symptoms reported might be those of late evolution of endocarditis.

Half the patients in our study already had damaged valves at presentation. Interestingly, no prior valvular disease was documented for most of the published patients with *B*. *quintana* endocarditis [4, 42, 46]. However, some studies reported rheumatic heart disease as the risk factor most frequently associated with *B*. *quintana* IE [15, 50, 53]. This may reflect differences in the clinical course of the disease between the Southwest Indian Ocean region and Europe, where studies have suggested that *B*. *quintana* IE followed chronic bacteremia in patients without prior valve defects [42]. Moreover, the mitral valve was the valve most frequently affected (75%) in our patients, whereas the aortic valve was the valve most frequently affected in published cases [57]. One hypothesis could be an association between previous presence of rheumatic heart disease (41.7%) in the cohort, and mitral valve affect. A predominant mitral valve involvement has already been observed in cases reporting rheumatic heart disease in medical history [15, 50, 53].

The recommended treatment for *B*. *quintana* endocarditis according to European guidelines is oral doxycycline (100 mg/12 h) for four weeks plus gentamicin (3 mg/24 h) for two weeks [19]. All the patients in our cohort received at least one of these two antibiotics. However, three patients were not treated with the recommended double-therapy regimen: patients 6, 9 and 12 (patients 5 and 10 received gentamicin for 2 weeks before *Bartonella* infection diagnosis, as initial antibacterial treatment). They received doxycycline alone, or associated to amoxicillin or rifampicin. Although not in accordance with the guidelines, theses regimens showed clinical success. Gentamicin is known to cause renal toxicity, nevertheless, this complication has not been observed in the cohort following the use of this antibiotic. *Bartonella* IE is known to cause significant destruction of valve tissues, necessitating valve surgery more frequently than IE caused by other pathogens [58]. Our study confirmed these findings, with all 12 cases requiring surgical treatment. This greater need for surgical treatment may be due to delayed management or a pre-existing altered state potentially aggravated by undernutrition and poor socioeconomic conditions and underlying the higher rates of postoperative complications (66.7%) than for other types of bacterial IE [59].

BCNE is associated with a higher rate of in-hospital adverse events than blood culture-positive endocarditis, although recent publications have suggested similar long-term outcomes [60]. Two patients developed postoperative mediastinitis. Only one other case has ever been reported to develop post-sternotomy mediastinitis following *B*. *quintana* IE [44]. Despite the short-term complications observed, none of the patients died, contrasting with the previous estimate of 7–30% for *Bartonella* IE-related mortality [26]. Patient 2 developed recurrent IE caused by *Streptococcus* spp. months and years after initial *B*. *quintana* IE diagnosis. This case highlights the risk factor that chronic *B*. *quintana* IE represents for additional endocarditis, and the possibility of underestimating *B*. *quintana* if the infection was concomitant. This has already been described by Boodman *et al*. who mentioned “subacute *B*. *quintana* infection creates valvular damage and large vegetations, which provide a substrate for seeding when acute bacteremia occurs with a different pathogen” [30, 61]. Serological results were positive (IgG ≥1:100) in 81.8% of patients, slightly below the sensitivity of 91% estimated for IFA by Edouard *et al*. [5]. Nevertheless, serology guided diagnosis in 6 of 11 cases (54.5%), with IgG titers ≥1:800, the recommended cutoff for *Bartonella* IE diagnosis [5]. Serological assay showed low performance in this study, compared to other studies that show a sensitivity of 63–94% [5, 47]. Maurin *et al*. has already showed the low sensitivity of the IFA-Focus Diagnostics kit in detecting *B*. *quintana* IgG on *B*. *quintana* endocarditis patients [22]. In addition, we noted that patient 11 presented a negative serology, possibly due to his severe undernutrition that could cause relative immunosuppression. Moreover, it is noteworthy the serological titers measured against both *B*. *quintana* and *B*. *henselae* cross-react between these two species, as already published [22, 27, 29, 33, 37, 47]. Also, recent studies have demonstrated cross-seroreactivity at low level between *Bartonella* spp. and other species in cases of blood culture positive endocarditis. Consequently, anti-*Bartonella* serology may be insufficient without confirmation by another technique, such as PCR in this study [47, 62]. PCR therefore remains the best diagnostic tool, provided that sufficient tissue is available. However, RT-PCR has a low sensitivity when applied to blood and serum samples (33% and 36%, respectively) [5]. Overall, molecular assays on tissues and serological testing perform well for the diagnosis of *B*. *quintana* IE. An increase in C-reactive protein concentration and anemia were observed in all patients. The anemia could be attributed to endocarditis-related inflammation. Thus, given that confirmation analyses can take a long time, we believe that any patient from Mayotte or Comoros presenting with symptoms of endocarditis, a biological inflammatory syndrome, and vegetations detectable on an echocardiogram but with negative blood cultures should be considered a possible case of *B*. *quintana* (or *Coxiella burnetti*) IE, until proven otherwise. In such cases, the early administration of doxycycline, before confirmation of the diagnosis, may be beneficial.

The *B*. *quintana* genotype circulating in the Southwest Indian Ocean region appears to be different from those circulating in Europe and Africa. Interestingly, the MST 6 genotype identified in our cohort was previously identified only once, in aortic valve tissues from a patient with *B*. *quintana* endocarditis in Australia [51]. All three strains successfully analyzed by MST had identical sequence profiles, and two of these strains were isolated two years apart. Despite the very small population analyzed, these findings suggest that there may be only one *B*. *quintana* genotype (MST 6) circulating in the Comoros archipelago, or that there is a potential common source of contamination on Mayotte. This exposure factor has yet to be identified. One of these patients was a farmer who may have come into contact with animals and lived in a rural community. The known risk factors for *B*. *quintana* endocarditis are homelessness, alcohol dependence and exposure to body lice [1]. It is reasonable to assume that these markers are linked to poor socioeconomic status [4]. Exposure to head lice was identified only in two cases, but we believe that information about head louse infestation history were missing, probably because patients had been infected months or years before their admission to hospital for IE. Moreover, Sangaré *et al*. demonstrated the effective presence of *B*. *quintana* vectors in the Southwest Indian Ocean area (in 4.5% of body and 2.6% of head lice in Madagascar, with even higher rates in other African countries, including East African countries) [63]. Furthermore, all of the patients studied here originated from Mayotte or Comoros, where a high proportion of the population has an income below the threshold defining the national poverty line and there are many exchanges of population with Southern Africa [64]. However, one of the limitations of this retrospective study is that the bacterial diagnosis was established a long time after admission. It was therefore not possible to identify and record possible sources of infection for most of the cases.

## Conclusion

We report here an original series of 12 cases of *B*. *quintana* IE from the Southwest Indian Ocean, where this disease has never before been described. Our data suggest that *B*. *quintana* is not an uncommon cause of native valve endocarditis in Comoros archipelago, particularly on Mayotte. Moreover, immigration from the Comoros archipelago to the French islands in this region is continuing, rendering this case series of particular interest. Local epidemiological and seroprevalence studies are required to determine the frequency of *B*. *quintana* infection in the Southwest Indian Ocean area. This study should raise local awareness of this neglected disease. *B*. *quintana* IE should be suspected in patients from Mayotte or Comoros with mitral valve dysfunction and blood culture-negative endocarditis, even in the absence of the traditional risk factors for *Bartonella* infection.

## Supporting information

S1 Table Multispacer typing (MST) genotyping results. (DOCX)
