# Supplementary material for: Case series of 12 Bartonella quintana endocarditis from the Southwest Indian Ocean
Source: PLoS Negl Trop Dis. 2023 Sep 7;17(9):e0011606. doi: 10.1371/journal.pntd.0011606 (PMC10508605; doi:10.1371/journal.pntd.0011606)
Supplement: S1 Table — (DOCX) [file pntd.0011606.s001.docx]

**Supporting information**

**S1 Table.** **Multispacer typing (MST) genotyping results**

| **Pat. No.** | **Sample source** | **Ct PCR IHU** | **Sequence type for spacer:** | | **Genotype (MST)** |
| --- | --- | --- | --- | --- | --- |
|  |  |  | **336** | **894** |  |
| 8 | Mitral vegetation | 13 | 1 | 5 | 6 |
| 10 | Aortic valve | 26 | 1 | 5 | 6 |
| 12 | Aortic valve | 28 | 1 | 5 | 6 |
